# Complete Genome Sequence of the Hydrocarbon-Degrading Strain Achromobacter sp. B7, Isolated during Petroleum Hydrocarbon Bioremediation in the Valparaiso Region, Chile

**DOI:** 10.1128/MRA.01326-18

**Published:** 2018-11-15

**Authors:** Valentina Méndez, Lisette Hernández, Francisco Salvà-Serra, Daniel Jaén-Luchoro, Roberto E. Durán, Bárbara Barra, Beatriz Piñeiro-Iglesias, Edward R. B. Moore, Michael Seeger

**Affiliations:** a Molecular Microbiology and Environmental Biotechnology Laboratory, Department of Chemistry & Centro de Biotecnología Daniel Alkalay Lowitt, Universidad Técnica Federico Santa María, Valparaíso, Chile; b Department of Infectious Diseases, Sahlgrenska Academy, University of Gothenburg, Gothenburg, Sweden; c Centre for Antibiotic Resistance Research (CARe), University of Gothenburg, Gothenburg, Sweden; d Culture Collection University of Gothenburg (CCUG), Sahlgrenska Academy, University of Gothenburg, Gothenburg, Sweden; e Microbiology, Department of Biology, University of the Balearic Islands, Palma de Mallorca, Spain; Queens College

## Abstract

Achromobacter sp. strain B7 (= CCUG 72081) was isolated from a diesel-polluted soil from the Valparaiso Region, Chile, subjected to bioremediation with a hydrocarbon-degrading enrichment. The complete genome sequence of Achromobacter sp. B7 has been determined to have a size of 6.24 Mb, 5,578 coding sequences, 57 tRNAs, and a G+C content of 64.8%.

## Abstract (precis)

Achromobacter sp. strain B7 (= CCUG 72081) was isolated from a diesel-polluted soil from the Valparaiso Region, Chile, subjected to bioremediation with a hydrocarbon-degrading enrichment.

## ANNOUNCEMENT

Achromobacter species are Gram-negative bacilli commonly found in soil and water but also are associated with human clinical samples. Relevant species include Achromobacter piechaudii, Achromobacter spanius, and Achromobacter marplatensis (1, 2). Here, we report the complete genome sequence of a hydrocarbon-degrading strain, Achromobacter sp. B7, isolated during bioremediation trials of a diesel-spiked soil from Laguna Verde, Valparaiso Region, Chile. Bioremediation involved the addition of a hydrocarbon-degrading bacterial enrichment (3). Achromobacter sp. B7 is able to grow in M9 minimal medium on hexane, octane, hexadecane, naphthalene, biphenyl, and diesel at 30°C (4).

Genomic DNA was isolated from a fresh culture biomass of Achromobacter sp. B7 grown on tryptic soy agar (TSA) and lysed in EDTA-saline buffer (0.15 M NaCl, 0.01 M EDTA, pH 8.0) with 10 mg ml^−1^ lysozyme for 2 h at 37°C. Genomic DNA was isolated with the Promega Wizard kit and a modified Marmur procedure (5). DNA was sequenced on an Illumina HiSeq 4000 system (GATC Biotech, Germany), generating 10,102,770 pair-end reads of 150 bp each and yielding a total of 1,525.5 Mb. Standard genomic library preparation was performed with an optimized protocol, and standard Illumina adapter sequences were used. Sequence reads were trimmed, using Sickle (version 1.33) (6), with a Phred quality score threshold of 30. A subset of 6,666,666 high-quality paired-end reads with a total of 904,208,607 bp was obtained. Additionally, DNA was sequenced with an Oxford Nanopore Technologies (ONT) MinION instrument. A library was prepared with the ONT rapid barcoding kit (SQK-RBK004). Albacore version 2.1.10 was used for base calling, which yielded a total of 2,260 Mb distributed in 217,643 reads. The subsets of Illumina paired-end reads (904 Mb) and base-called Nanopore reads (2,260 Mb) were used to perform a hybrid assembly, using hybridSPAdes from SPAdes version 3.11.1 (7, 8). The hybrid assembly resulted in a final closed and complete chromosome sequence of 6,236,552 bp with a G+C content of 64.8%.

The B7 genome was annotated with the NCBI Prokaryotic Genome Annotation Pipeline (PGAP) version 4.6 (9), which identified 5,578 coding sequences and 57 tRNAs. After 16S rRNA gene sequence analyses, A. spanius DSM 23806 was the most closely related species (100% identity). Analysis by average nucleotide identity based on BLAST (ANIb) (10), using JSpeciesWS version 3.0.20 (11), with A. piechaudii NBRC 102461^T^, A. spanius DSM 23806 ^T^, and A. marplatensis sp. B2^T^, resulted in ANIb values of 88.0%, 87.4%, and 85.4%, respectively. Therefore, strain B7 most likely represents a novel species of Achromobacter. Genome analysis, with the Comprehensive Antibiotic Resistance Database 3.0.0 (CARD) (12), identified four antibiotic resistance genes in strain B7 (DVB37_25330, DVB37_12020, DVB37_10250, and DVB37_17655), possessing 82%, 42%, 43%, and 43% identity, respectively, with a resistance-nodulation-cell division (RND) efflux pump for fluoroquinolone and tetracycline. Gentisate (DVB37_25350) and homoprotocatechuate (DVB37_16865) dioxygenases possessed 39% and 63% identity with Streptomyces sp. strain WA46 and Escherichia coli C enzymes, respectively. Catechol-1,2-dioxygenase (DVB37_15220) and the protocatechuate-3,4-dioxygenase alpha subunit (DVB37_19170) possessed 42% and 79% identity with Achromobacter sp. strain ADP1 and Achromobacter lwoffii K24 enzymes.

The genome sequence of Achromobacter sp. B7 represents essential data for genomic and metabolic characterization of environmental Achromobacter strains.

### Data availability.

This whole-genome shotgun project has been deposited at DDBJ/ENA/GenBank under the accession number CP032084. The version described in this paper is the first version, CP032084.1. The accession number for the publicly available raw data at NCBI is PRJNA481776.
